# Multimodal data for predictive medicine: algorithmic fusion of clinical data in anesthesiology and intensive care

**DOI:** 10.3389/fmed.2026.1746867

**Published:** 2026-01-23

**Authors:** Sebastian Daniel Boie, Niklas Giesa, Maria Sekutowicz, Rustam Zhumagambetov, Stefan Haufe, Elias Grünewald, Felix Balzer

**Affiliations:** 1 Institute of Medical Informatics, Charité-Universitätsmedizin Berlin, Berlin, Germany; 2 Department of Anesthesiology and Operative Intensive Care Medicine (CCM, CVK), Charité - Universitätsmedizin Berlin, Corporate Member of Freie Universität Berlin, Humboldt-Universität zu Berlin, Berlin, Germany; 3 Berlin Institute of Health at Charité – Universitätsmedizin Berlin, BIH Biomedical Innovation Academy, BIH Charité Junior Clinician Scientist Program, Berlin, Germany; 4 Physikalisch-Technische Bundesanstalt, Berlin, Germany; 5 Technische Universitat Berlin, Berlin, Germany

**Keywords:** anesthesiology and intensive care, artificial intelligence, data science & machine learning, machine learning (ML), multimodal data fusion

## Abstract

Anesthesiology and intensive care medicine are among the most data-rich fields of medicine, where accurate and timely outcome prediction or risk stratification is important. During patient care, heterogeneous data streams, including structured electronic health records, free-text documentation, and high-frequency physiologic time series are recorded. This provides a fertile ground for machine learning (ML) models to make individualized risk predictions. Yet, secondary use of routine data remains difficult due to heterogeneity, missingness, variable granularity, ambiguously defined outcomes, or poor representation of clinical concepts in routine data. Reproducibility and transparency are difficult to achieve with hospital-specific complex data pipelines. New complexities arise when combining different data modalities. This perspective article discusses three common modalities—tabular data, clinical text, and time series—and outlines data modality-specific challenges, data preprocessing strategies, and ML modeling approaches. We examine multimodal fusion strategies through the common taxonomy of early, intermediate, and late fusion. In early fusion, generated features are aggregated into a unified tabular representation, offering simplicity and often serve as first baseline prediction models. Intermediate fusion uses modality-specific encoders with shared layers to learn cross-modal dependencies. This strategy yields the most complex and powerful models. Late decision-level fusion combines outputs from modality-optimized models, providing modularity and robustness to missing modalities, leading to advantages for real-time deployment where data arrive asynchronously. The growth of multi-centric datasets and federated infrastructures may enable intermediate-fusion architectures and multimodal foundation models to better capture patient trajectories, supporting risk stratification and personalized therapy in perioperative and intensive care settings.

## Introduction

Patients in anesthesiology and intensive care units are among the most closely monitored patients. Different data types, such as continuous physiologic signals, high-frequency device outputs, and detailed electronic health records (EHRs), produce large streams of data (1). Traditional clinical decision-making tools, which are based on guidelines and physician expertise are increasingly challenged by addressing the volume and complexity of information available today. This environment has created opportunities for the development of machine (ML) applications that promise to reshape perioperative and intensive care medicine. Typical use cases for ML are perioperative risk prediction for complications such as acute kidney injury (2, 3) and post-operative delirium (4, 5), real-time physiologic monitoring and early-warning systems for hypotension and deterioration (6, 7) or early detection and outcome prediction in sepsis (8).

It is well-known that research based on routine data is challenging due to complexity, heterogeneity, and data quality challenges. Clinical and administrative data is primarily collected for coordinating care and billing, so use for other purposes may lead to difficulties. Clinical workflows are typically not optimized for research-grade data quality. Inconsistencies, missingness, and varying levels of granularity across institutions are equally challenging (9).

Standardization is a large area of research that alleviates some of the pain points. The major standardization efforts are the Observational Medical Outcomes Partnership (OMOP) common data model for standardized storage of observational data (10), Fast Healthcare Interoperability Resources (FHIR) for standardized data exchange (11), the openEHR framework (12) for standardized clinical information representation and HL7 Structured Data Capture (SDC) for standardized data capture.

Missingness patterns are categorized into missing completely at random (MCAR), where missingness is independent of both observed and unobserved values, missing at random (MAR), where missingness is only dependent on observed values, and missing not at random (MNAR), where missingness is dependent on unobserved values. Typically, all three patterns are observed in routine data, and assumptions about missingness are generally untestable in practice, requiring sophisticated strategies for imputation by estimating missing values on a training cohort (13–19).

Missingness of outcomes is a bigger hurdle than missing predictors. Reliable definitions of outcomes are challenging for many outcomes of interest, as endpoints are sometimes observed with delay (right-censored at prediction time), ambiguously defined or insufficiently captured. Therefore, formulating clinically meaningful prediction tasks and developing robust models are challenging. Many researchers default to predicting in-hospital mortality or length of stay (20), even though the prediction is rarely actionable, since it’s not specific enough to suggest an intervention and the cause can seldomly be addressed over a short period of time.

Virtually every treatment episode in anesthesiology and intensive care results in tabular data (e.g., demographic factors, billing information, comorbidities, laboratory results, medication administration), free-text data (e.g., anamnesis, admission and discharge notes) and time-series or waveform signals from monitors and devices (e.g., heart rate, blood pressure) (21). Some patients also have recorded imaging data (e.g., CT, MRI or X-ray) during their treatment episode. Emerging data sources, such as genomics data, wearable data and patient-reported outcomes fit into the existing modalities of tabular, text and time series data.

Combining different data modalities complicates integration and joint analysis. The data modalities are often recorded at irregular intervals (from multiple samples a second for waveform data to daily documentation of risk scores and observations), requiring sophisticated preprocessing and alignment methods (22, 23).

In this article, we will discuss the three common data modalities (tabular data, clinical notes and time series data) highlight challenges and common approaches to analyze each modality and discuss typical approaches for a joint analysis of modalities for predicting outcomes.

## Data modalities and analysis strategies

The three key data modalities tabular data, text, and time series may contain overlapping information, but provide distinct insights that are essential for comprehensive patient monitoring and outcome prediction. In this perspective, we focus on the three modalities that are available for every patient in anesthesiology and intensive care.

### Tabular data

Tabular data is information organized in predefined fields and formats, organized in tables or key-value pairs, such that each variable has consistent meaning and data type. Data types can be continuous or categorical.

Common data quality issues of tabular data are the use of different physical units (e.g., height in m or cm) and extreme values or outliers (24). Each datum has an associated timestamp; however, these timestamps can be inaccurate (e.g., due to miscalibrated clocks, daylight saving time adjustments, or diagnoses and procedures being coded only at the end of the hospital stay for billing) (25). To mitigate these quality issues, manual review of variables to check consistent units followed by mapping to a standard vocabulary and unit, applying reasonable bounds and robust scaling like z-score standardization have proved effective (26, 27).

A vast body of literature is dedicated to outcome prediction based on tabular data (2, 28–30). Unlike for other data modalities, there is no *a-priori* correlation structure to exploit. For other data modalities, specific architectures (e.g., CNNs for images, recurrent NNs or Transformer for time series and text) can capitalize on the inherent correlation structure in these modalities. However, correlations exist (e.g., between height, weight and diagnoses codes coding obesity) and can be learned by models during the training process.

Models for prediction based on tabular data range from simple models like linear (continuous prediction) or logistic regression (categorical prediction) to advanced algorithms like tree-based methods (e.g., xgboost), deep neural networks (e.g., multi-layer perceptrons) and lately neural-network based foundation models (31). The widely used xgboost algorithm is often a very strong candidate for high prediction accuracy (32).

### Free-text

A substantial part of relevant information is documented in free text (e.g., anamnesis, progress notes). The advantage of free text is that information that does not fit into a predefined tabular format can be documented. For instance, information like clinical reasoning and interpretation of data (e.g., a patient is hypotensive likely due to septic shock) and rationale (e.g., increased norepinephrine due to persistent low MAP) is typically contained in the clinical notes. Free text also allows for detailed patient history documentation, event descriptions, care coordination, nursing observations and other contextual information not captured in tabular data (33, 34). Furthermore, free-text is often preferred by experienced clinicians as it is usually considered to be more practical and time-efficient for data entry as opposed to structured data input.

Analyzing free-text data is arguably more challenging compared to other data types. While implicit standards for clinical notes exist, no rules are enforced, since conditions can be described in many ways, local customs have become established, or the clinical information systems limit their expressiveness. Free-text can be context dependent on other data modalities, contain a mix of tabular (e.g., a specific lab value) and word documentation, non-standard abbreviations, jargon, typos, and multilingual entries (35). The secondary use of free-text data for research is also hampered by legal and privacy considerations since personally identifiable information can be included and is challenging to remove in an automated fashion.

Traditionally, free-text data has been included in predictive models by mapping elements to tabular data using a standardized terminology such as SNOMED CT (36) or by extracting relevant information using either a rule-based or model-based strategies such as named-entity-recognition (37) and bag-of-words (38). Nowadays, there is a growing interest in working with free-text data that automatically converts text to relevant representations for prediction using deep-learning approaches (e.g., BioBERT, ClinicalBERT, MedPaLM 2) (39, 40).

### Time series/waveforms

Continuously monitored and recorded parameters are often stored as time series. Since typical parameters (e.g., heart rate, respiratory rate) exhibit periodic patterns, they are also called waveforms. This modality uniquely captures dynamic changes in biosignals of individuals.

These data are not free of artifacts due to sensor disconnection or misplacement, motion artefacts, data transmission errors or procedures initiated by the hospital staff. Artifacts can include missing or extreme values beyond physiologically plausible limits. Often a simple last-observation carried forward imputation is therefore performed, which has been shown to be competitive for time series data (41).

High frequency time series exhibit unique challenges. Some signals are recorded with 500 samples per second and quickly generate millions of data points per patient and day. Analyzing such data for an entire cohort requires specialized (distributed) algorithms for compression, indexing, storage, and retrieval (42). Common basic approaches are low-pass filtering and downsampling (43). Additionally, detecting signals pertaining to the onset of some event of interest is akin to finding the needle in the haystack.

Traditionally, time series are incorporated into prediction models by aggregation strategies that collapse the signal over a predefined period of time (44). Such strategies are easy to implement and allow researchers to use same models as for tabular data. However, this approach discards much of the temporal structure. Alternatively, modern approaches include algorithms that process raw time series data directly (e.g., recurrent neural networks that read one value per feature per time step) (45).

### Combining data modalities

Each of the three data modalities has its own challenges, mitigation strategies, and routinely used algorithms for analysis. While many studies have used multiple modalities for outcome prediction, this is often done by a simple integration strategy neglecting the inherent structure of some modalities. This possibly discards information useful for solving prediction tasks (44, 46).

**Figure 1:**
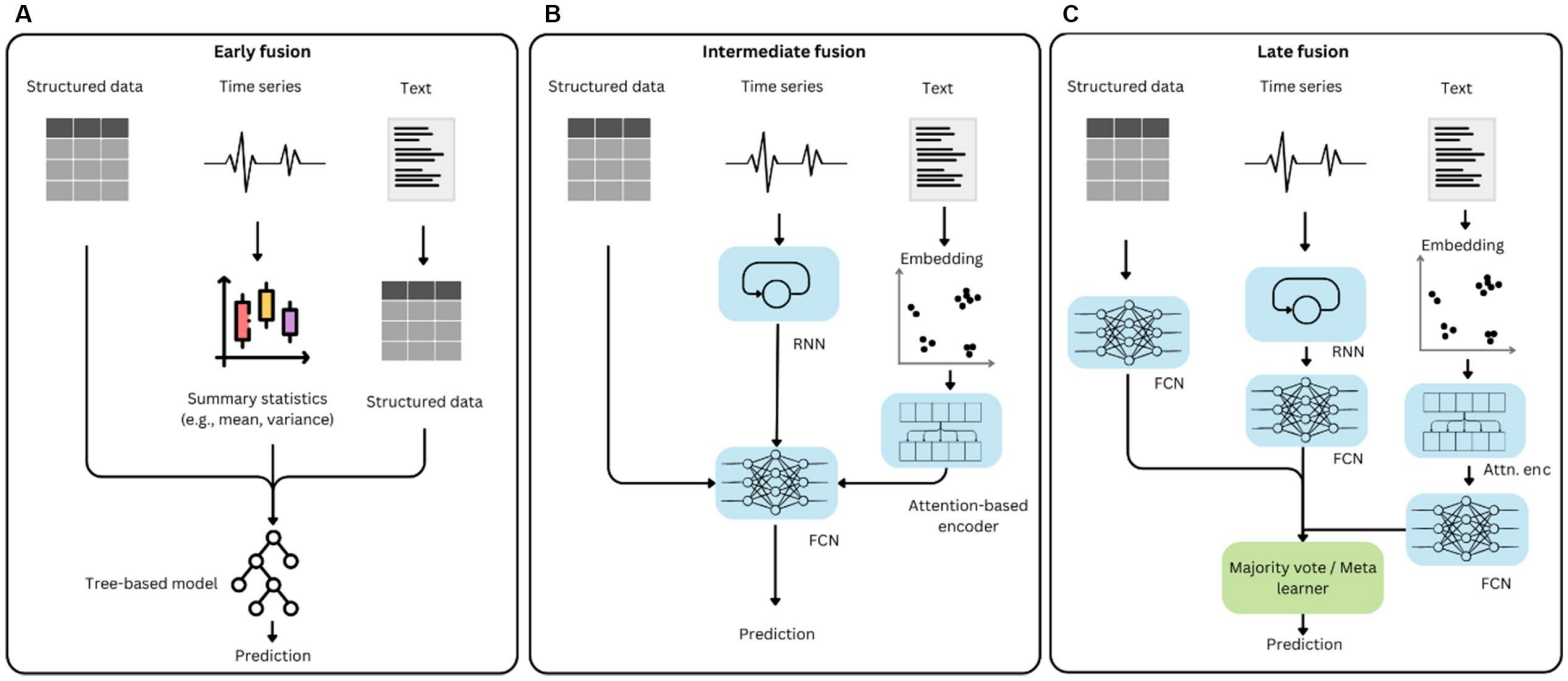
Data modality fusion strategies. The left panel **(A)** shows an example of early fusion, where data modalities are combined prior to model input. The middle panel **(B)** shows an example of intermediate fusion, where data of different modalities are ingested by bespoke algorithms before being combined by a central model. The right panel **(C)** shows an example of late fusion, where each modality is used independently in an appropriate model, while only model outputs are combined at the decision stage.

In the ML community, there is a widely-used taxonomy of early fusion, intermediate fusion, and late fusion of different data modalities for addressing machine learning tasks (47). We discuss this taxonomy in the context of outcome prediction in anesthesiology and intensive care. An overview of the fusion strategies can be seen in Figure 1.

### Early fusion

Early fusion combines heterogeneous data at the input level. Commonly, this is done by using tabular data directly, and by extracting features from time series and text (see Figure 1A). Features from time series can be obtained by manually extracting summary statistics (e.g., mean, variance, percentiles) across the entire time series or segments of it. This hand-crafted feature selection is often guided by domain knowledge therefore needs input from experts (48). Alternatively, there are solutions for automatically extracting hundreds of time series features based on hypothesis testing procedures (49).

In early fusion, text is often incorporated by searching for keywords, lexicon or regular expression-based matching (50). This form of information extraction leads to a tabular documentation of relevant entities which can then be used downstream in the prediction task. For these tasks, a range of libraries are available (e.g., spaCy, NLTK, MedCAT) (51, 52). Features are also commonly obtained via embedding models that produce dense vector representations of text.

In early fusion, these representations are concatenated with tabular data and time-series features to form a single, large feature vector, which serves as input to a downstream prediction model. Prediction models that operate on modalities fused in this way are discussed in the tabular data section. However, early fusion can discard important information from individual modalities, potentially reducing performance on downstream prediction tasks.

### Intermediate fusion

A joint analysis of tabular, textual, and time-series data substantially increases methodological complexity. It requires aligning heterogeneous data modalities with different temporal resolutions, standardizing variable definitions across modalities, and resolving inconsistencies in documentation practices.

Intermediate fusion integrates data at the representation level. Here, each modality is first processed by a modality-specific encoding model that transforms raw input into a latent feature representation. The encoded representations are then combined in a shared architecture, typically using concatenation, attention, or gating mechanisms that allow the model to learn cross-modal interactions (53, 54).

A common setup involves neural encoders tailored to each modality—for instance, feed-forward networks for tabular data, pre-trained language models such as BERT or ClinicalBERT for text, and Transformer or recurrent architectures for time series (55, 56). The encoded data are then fused in a joint layer, projecting into a shared latent space, where information can be attended to across modalities. A key feature is that the entire architecture, from joint layer to individual modality encoders, can be jointly trained or fine-tuned, such that all components are optimized towards the prediction task of interest. This supports flexible and data-driven representation learning and has become one of the most widely used and powerful strategies in multimodal machine learning (55, 57).

Intermediate fusion offers several advantages over early and late fusion. It allows each modality to contribute learned features rather than relying on handcrafted summaries, and it can discover complex nonlinear dependencies between modalities. However, it typically requires more computational resources and training data, and careful design choices are needed to balance the contribution of each modality, to prevent overfitting, and to handle missing modalities during inference (58).

Recent work in clinical prediction models has demonstrated high performance of intermediate fusion architectures. For example, cross-modal attention and gating mechanisms have been successfully applied to integrate physiologic time series with clinical notes and tabular EHR data, yielding improvements in mortality and event prediction tasks (59). Such architectures illustrate the potential of data-driven representation learning to capture intricate patterns of different data modalities.

### Late fusion

Late fusion (or decision-level fusion) combines the outputs of separate models trained independently on individual data modalities, for example through weighted averaging, majority voting or stacking. Each modality is modeled using an algorithm most suitable given its characteristics. The predictions are then combined in a decision layer (see Figure 1C). This architecture offers practical advantages. It is modular by design, and individual components can be retrained or replaced without affecting other parts. Late fusion is robust to missing modalities, since predictions are still generated from available data modalities. Therefore, it is particularly applicable to real-time ML in intensive care, where waveform data may be unavailable during certain procedures and textual documentation may occur only after care decisions are taken and executed. Additionally, with this architecture, some interpretability is given by the fact that it becomes apparent which modality and set of clinical parameters lead to a certain prediction.

The major shortcoming of this strategy is that it cannot learn complex interdependencies between data modalities (e.g., a co-occurrence of an abnormal lab value (tabular), an unusual blood pressure pattern (time series) and a documented observation which may be inconclusive individually but very informative taken together).

Table 1 shows a brief summary of the different fusion strategies with advantages, disadvantages and examples.

**Table 1:** Summary table of data fusion strategies with advantages, disadvantages and examples.

Fusion strategy	Fusion level	Advantages	Disadvantages	Examples
Early	Feature	Simple approach, often uses human interpretable features	Discards possibly relevant information during aggregation	Keyword extraction + summary statistics + tabular data
Intermediate	Representation	Can learn patterns across data modalities jointly	Requires complex architecture, resource intensive	Combined architecture using recurrent NN for time series transformers for text and MLP to fuse modalities
Late	Decision	Modular approach, easy to change models for modalities, easy to handle missing modality	No learning across data modalities	Ensemble models that make decision on single modalities

## Clinical perspective

In this perspective article, we discuss key challenges, strategies and architecture design patterns for three common data modalities in anesthesia and intensive care. With complementary data modalities available during different phases of clinical care, the multimodal integration holds substantial potential to enhance understanding of disease trajectories, and to improve ML predictions to support clinical decisions, stratify risk groups, and enable personalized therapy (57, 58).

In anesthesiology and intensive care medicine, tabular, text and time series data are commonly acquired for every patient. Each of these modalities comes with distinct challenges and requirements for preprocessing strategies (60). Multimodal integration demands robust strategies for harmonizing units, ontologies, and timestamps, as well as methods that can gracefully handle incomplete records or entirely missing modalities.

The multimodal ML community has a widely used taxonomy of data fusion strategies, which is directly applicable to ML use cases in anesthesia and intensive care. Since each fusion strategy has advantages and limitations, it is important to carefully consider which strategy is chosen for a given prediction task. We provide guidance below.

Early fusion is simple and powerful when reliable feature extraction strategies exist (e.g., curated time-series summaries and keyword/entity based textual features). This approach requires less computational resources than other strategies and less training data. Hence, it is particularly suitable for smaller data sets and single-center studies.

Intermediate fusion (encoder-specific layers for each modality with shared fusion layers) best captures cross-modal interactions and has the potential to yield the strongest predictive performance among strategies (61). It puts higher computational requirements on the model development and requires larger representative data sets. In the future, with national and international data sharing and federated machine learning infrastructures being developed, intermediate fusion models are likely to attain superior performance (58).

Late fusion is modular, robust to missing data modalities, and suitable for real-time deployment where modalities arrive asynchronously. While it cannot learn complex cross-modal dependencies, its strength lies in fault-tolerant architectures with easier-to-achieve real-time capabilities, making this strategy especially suitable for first production pilots.

Machine learning-based (multimodal) predictions are valuable only if they support actions or inform processes. Prediction tasks should be defined by the decisions they enable for interventions in a given time horizon (e.g., vasopressor titration, fluid administration, antibiotic escalation, ICU triage). Outcome choices, such as in-hospital mortality or length of stay, are comparably easy to label but rarely actionable. Designing prediction tasks and evaluation strategies is one of the primary challenges in clinical ML (62). The availability of multiple data modalities adds complexity but will likely yield the highest performance.

Recent multimodal studies illustrate these considerations. Benchmarking work in emergency settings has used late fusion to combine high-frequency waveforms with structured clinical data, demonstrating added value of waveform signals for acute risk stratification (63). Sepsis trajectory models using multivariate temporal EHR data employ early fusion to encode longitudinal predictors and improve continuous risk estimation (64).

Also, studies predicting postoperative deterioration or cardiac events from combined waveform and EHR data largely adopt early fusion, integrating engineered waveform features with structured variables before modeling (65, 66). In deployment contexts, intraoperative hypotension-prediction systems such as the Hypotension Prediction Index exemplify unimodal early fusion of high-frequency waveform features for real-time decision support (6).

Collectively, these studies show that multimodal fusion strategies are already used in clinical research and practice, reinforcing the potential of multimodal ML to enhance real-time risk assessment in emergency and perioperative care.

## Conclusion

Multimodal ML applied to tasks in anesthesiology and intensive care is an emerging field with large potential. Early fusion is usually the fastest path to a baseline prediction, late fusion is the most resilient architecture for real-time deployment, and intermediate fusion offers the highest predictive performance. With larger multi-centric datasets and national and international federated infrastructures being developed, multimodal foundation models with an intermediate fusion strategy may prove to be transformative for personalized medicine.

## Data Availability

The original contributions presented in the study are included in the article/supplementary material, further inquiries can be directed to the corresponding author.
